# Extent of resection and adjuvant radiotherapy in intracranial anaplastic meningioma: improved disease control with no statistically significant overall survival benefit

**DOI:** 10.3389/fsurg.2026.1841444

**Published:** 2026-09-16

**Authors:** Yanming Miao, Haibin Wan, Xinyan Zhao, Weifeng Jia, Liangning Shui, Miao Zong, Dabiao Zhou

**Affiliations:** 1 Department of Neurosurgery, Beijing Electric Power Hospital, Beijing, China; 2 Capital Medical University Electric Power Teaching Hospital, Beijing, China; 3 Department of Neurosurgery, Beijing Tiantan Hospital, Capital Medical University, Beijing, China

**Keywords:** anaplastic meningioma, progression-free survival, radiotherapy, simpson grading, tumor recurrence

## Abstract

**Background:**

Intracranial anaplastic meningioma, currently classified as World Health Organization (WHO) Grade III, is a rare and biologically aggressive tumor with a high recurrence rate. Due to its rarity, the available literature on its clinical characteristics and prognostic factors remains limited. In this study, we aimed to evaluate the relative impact of the extent of surgical resection and adjuvant radiotherapy on long-term survival and recurrence control in patients with intracranial anaplastic meningioma.

**Methods:**

We conducted a retrospective cohort study of patients with histopathologically confirmed World Health Organization (WHO) Grade III meningioma. Among 5,962 cases treated between January 2012 and July 2022, 46 patients with complete follow-up data were included. Clinical characteristics, treatment modalities, and outcomes were analyzed. Kaplan–Meier analysis was used to assess progression-free survival (PFS) and overall survival (OS).

**Results:**

During follow-up, 38 patients (82.6%) experienced tumor recurrence. The median PFS was 24 months, with 2- and 5-year recurrence rates of 52.0% and 77.0%, respectively. The median OS was 68 months, with 2- and 5-year OS rates of 79.7% and 56.5%. Simpson Grade I/II resection was associated with longer OS than Grade III/IV resection, although the difference was not statistically significant. Adjuvant radiotherapy showed a trend toward improved OS but did not reach statistical significance. In contrast, radiotherapy was associated with delayed recurrence and prolonged PFS, particularly in terms of early recurrence control.

**Conclusions:**

Intracranial anaplastic meningioma is characterized by high recurrence and poor prognosis. In this cohort, neither extent of resection nor adjuvant radiotherapy reached a statistically significant improvement in overall survival, whereas both were associated with delayed tumor recurrence. These findings suggest that current treatment strategies may primarily provide temporal disease control rather than substantially altering long-term survival outcomes.

## Introduction

Anaplastic meningioma [World Health Organization (WHO) Grade III] is a rare yet highly aggressive subtype of meningioma (1). It is distinguished by pronounced cellular atypia, heightened mitotic activity, necrosis, and brain invasion (1, 2). According to the WHO classification of central nervous system tumors, anaplastic meningiomas constitute approximately 1%–3% of all meningiomas (3). Despite their low incidence, these tumors are linked to significantly poorer clinical outcomes when compared with benign or atypical meningiomas (4).

Surgical resection remains the fundamental treatment for anaplastic meningioma. Repeated Gamma Knife stereotactic radiosurgery at the time of progression can offer long-term survival for these patients. However, the inherent biological aggressiveness of these tumors leads to a significantly increased risk of recurrence and mortality. Previous studies have reported that the recurrence risk is six- to eight-fold higher than that of lower-grade meningiomas (5–7). Even after gross total resection combined with adjuvant radiotherapy, recurrence is still common, and long-term survival outcomes remain suboptimal.

Radiotherapy, encompassing fractionated external beam radiation therapy and stereotactic radiosurgery, is widely employed as an adjuvant or salvage treatment modality (8). Current clinical guidelines recommend routine postoperative radiotherapy for WHO Grade III meningiomas (9). Nevertheless, the consistently high recurrence rate and limited long-term therapeutic efficacy suggest that current treatment strategies are still inadequate.

In recent years, advancements in molecular profiling have enhanced our comprehension of the genomic and epigenetic landscape of high-grade meningiomas, presenting potential opportunities for targeted therapies and immunotherapeutic approaches (10). However, these strategies are largely in the investigational stage, and their clinical application has yet to be fully established.

Therefore, further studies are necessary to optimize treatment strategies, refine prognostic stratification, and identify patients who may benefit most from aggressive multimodal therapy. In the present study, we retrospectively analyzed a cohort of patients with histologically confirmed intracranial anaplastic meningioma treated at two high-volume neurosurgical centers. We aimed to assess the impact of surgical extent (Simpson grading) and adjuvant radiotherapy on tumor recurrence, progression-free survival (PFS), and overall survival (OS). Our findings offer additional insights into the clinical behavior of anaplastic meningioma and may assist in informing contemporary management strategies.

## Materials and methods

### Patient selection and data collection

This retrospective dual-center cohort study included patients with histopathologically confirmed intracranial anaplastic meningioma [World Health Organization (WHO) Grade III] treated at the Department of Neurosurgery, Beijing Tiantan Hospital and Beijing Electric Power Hospital between January 2012 and July 2022.

During the study period, a total of 5,962 patients underwent surgical treatment for intracranial meningiomas. Among them, 63 patients were diagnosed with WHO Grade III anaplastic meningioma. Inclusion criteria were: (1) histopathological confirmation of intracranial anaplastic meningioma; (2) surgical treatment at one of the participating institutions; and (3) availability of complete clinical, radiological, and follow-up data. Exclusion criteria included: (1) loss to follow-up; (2) perioperative mortality; and (3) receipt of non-standard initial radiosurgical treatment, such as Gamma Knife radiosurgery without conventional external beam radiotherapy.

After applying these criteria, 46 patients were included in the final analysis. The cohort consisted of 26 males and 20 females, with a mean age of 46 years (range, 1–74 years). A total of 108 surgical procedures were performed during the overall disease course, reflecting multiple surgeries in patients with recurrent disease.

Comprehensive clinical data were collected retrospectively from institutional medical records, including demographic characteristics, presenting symptoms, tumor location, radiological findings, extent of resection, adjuvant treatment, pathological features, and long-term outcomes.

### Imaging and histopathological evaluation

All patients underwent standardized preoperative neuroimaging, including computed tomography (CT) and contrast-enhanced magnetic resonance imaging (MRI). Radiological assessment included tumor location, size, enhancement pattern, peritumoral edema, and evidence of invasion into adjacent brain tissue or dural structures.

Typical imaging features of anaplastic meningioma included irregular tumor margins, heterogeneous contrast enhancement, hyperintensity on diffusion-weighted imaging (DWI), and extensive peritumoral edema (Figure 1).

**Figure 1:**
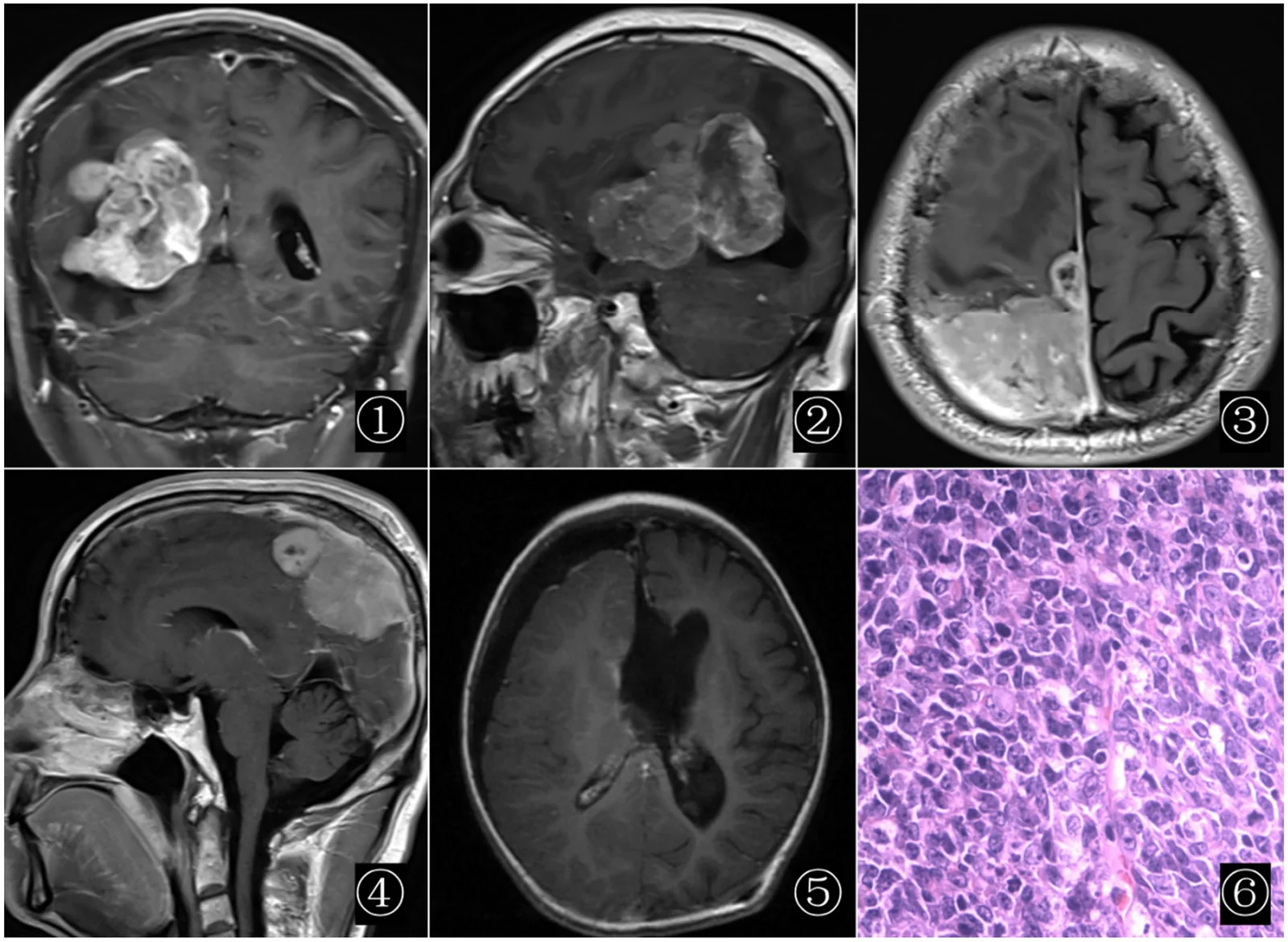
(1, 2) Axial and coronal magnetic resonance imaging (MRI) depicts a tumor situated in the trigone of the lateral ventricle. The tumor presents a cauliflower-like morphology, with heterogeneous enhancement and focal necrosis. (3, 4) Axial and sagittal views illustrate a right parietal lobe meningioma that invades the sagittal sinus. The meningioma has irregular margins and shows homogeneous contrast enhancement. (5) Postoperative imaging reveals that a tumor in the third ventricle has been resected to the Simpson Grade I extent. (6) Hematoxylin and eosin (HE) staining at 200× magnification confirms the diagnosis of anaplastic (WHO Grade III) meningioma.

Histopathological diagnosis was established according to the latest WHO classification criteria for central nervous system tumors. Immunohistochemical analyses included assessment of Ki-67 labeling index (LI), p53 expression, transforming growth factor-alpha TGF-α, and epithelial membrane antigen (EMA), when available.

### Surgical management and adjuvant radiotherapy

All patients underwent microsurgical tumor resection performed by experienced neurosurgeons with the goal of achieving maximal safe resection while preserving neurological function. Surgical planning was based on tumor location, radiological characteristics, and involvement of critical neurovascular structures. Neuronavigation and intraoperative neurophysiological monitoring were used when clinically indicated.

Extent of resection was classified according to the Simpson grading system. Gross total or near-total resection (Simpson Grade I/II) was achieved in 24 patients, whereas subtotal or partial resection (Simpson Grade III/IV) was performed in 22 patients.

Postoperative adjuvant radiotherapy was recommended whenever feasible. Among patients with Simpson Grade I/II resection, 14 received postoperative external beam radiotherapy, while 10 did not. In the Simpson Grade III/IV group, 17 patients underwent adjuvant radiotherapy and 5 did not. The mean radiation dose was 58 Gy, delivered in conventional daily fractions of 1.8–2.0 Gy.

Radiotherapy was not administered in some patients because of poor postoperative neurological condition, advanced age, treatment refusal, financial limitations, or rapid postoperative disease progression.

### Follow-up and outcome assessment

Patients were followed every 3 months during the first postoperative year and every 6 months thereafter. Follow-up evaluations included neurological examination and contrast-enhanced brain MRI.

The primary study endpoints were progression-free survival (PFS) and overall survival (OS). PFS was defined as the interval from initial surgery to radiological or clinical evidence of tumor recurrence or progression. OS was defined as the interval from initial diagnosis to death from any cause or last follow-up.

Tumor recurrence was determined based on serial radiological evidence of new or progressive enhancing lesions consistent with disease progression.

### Statistical analysis

Statistical analyses were performed using GraphPad Prism version 10.4.0 Continuous variables are presented as mean values with ranges, and categorical variables are presented as frequencies and percentages. Survival distributions for PFS and OS were estimated using the Kaplan–Meier method.

Comparisons between survival curves were performed using both the Mantel–Cox log-rank test and the Gehan–Breslow–Wilcoxon test to evaluate long-term and early survival differences, respectively. A two-sided *p* value < 0.05 was considered statistically significant.

## Results

### Patient characteristics

A total of 46 patients with histopathologically confirmed intracranial anaplastic meningioma were included in the final analysis. The cohort consisted of 26 males and 20 females, with a mean age of 46 years (range, 1–74 years). All patients underwent microsurgical resection followed by longitudinal radiological and clinical follow-up.

Gross total or near-total resection (Simpson Grade I/II) was achieved in 24 patients (52.2%), whereas subtotal or partial resection (Simpson Grade III/IV) was performed in 22 patients (47.8%). Postoperative external beam radiotherapy was administered to 31 patients (67.4%), with a mean radiation dose of 58 Gy delivered in conventional fractions. Fifteen patients did not receive postoperative radiotherapy because of poor postoperative condition, treatment refusal, advanced age, financial limitations, or rapid disease progression.

Detailed demographic and treatment-related characteristics are summarized in Table 1.

**Table 1:** Baseline clinical characteristics of patients with intracranial anaplastic meningioma.

Variable	Total Cohort (*n* = 46)
Age, mean (range), years	46 (1–74)
Sex	
Male	26 (56.5%)
Female	20 (43.5%)
Extent of resection	
Simpson Grade I/II	24 (52.2%)
Simpson Grade III/IV	22 (47.8%)
Postoperative radiotherapy	
Yes	31 (67.4%)
No	15 (32.6%)
Mean radiation dose	58 Gy
Tumor recurrence	38 (82.6%)
Death during follow-up	25 (54.3%)
Mean follow-up duration	72 months
Median progression-free survival	24 months
Median overall survival	68 months
Ki-67 labeling index (available cases)	47% (30%–80%)
p53 overexpression	74%
TGF-α overexpression	54%

### Histopathological and immunohistochemical findings

All tumors were confirmed as WHO Grade III anaplastic meningioma based on histopathological examination. Histological features included marked nuclear atypia, elevated mitotic activity, necrosis, and invasive growth patterns.

Immunohistochemical analysis demonstrated frequent overexpression of proliferative and oncogenic markers. Ki-67 labeling index (LI) was available in 9 patients and showed markedly elevated proliferative activity, with a mean value of 47% (range, 30%–80%). p53 overexpression was observed in 74% of tumors, whereas transforming growth factor-alpha TGF-α overexpression was identified in 54% of cases.

These findings support the highly aggressive biological behavior of intracranial anaplastic meningioma and suggest a potential association between elevated proliferative indices and tumor progression.

### Tumor recurrence and progression-free survival

During a mean follow-up period of 72 months, tumor recurrence was observed in 38 patients (82.6%). The median progression-free survival (PFS) for the entire cohort was 24 months. Kaplan–Meier analysis demonstrated cumulative recurrence rates of 52.0% at 2 years and 77.0% at 5 years (Figure 2).

**Figure 2:**
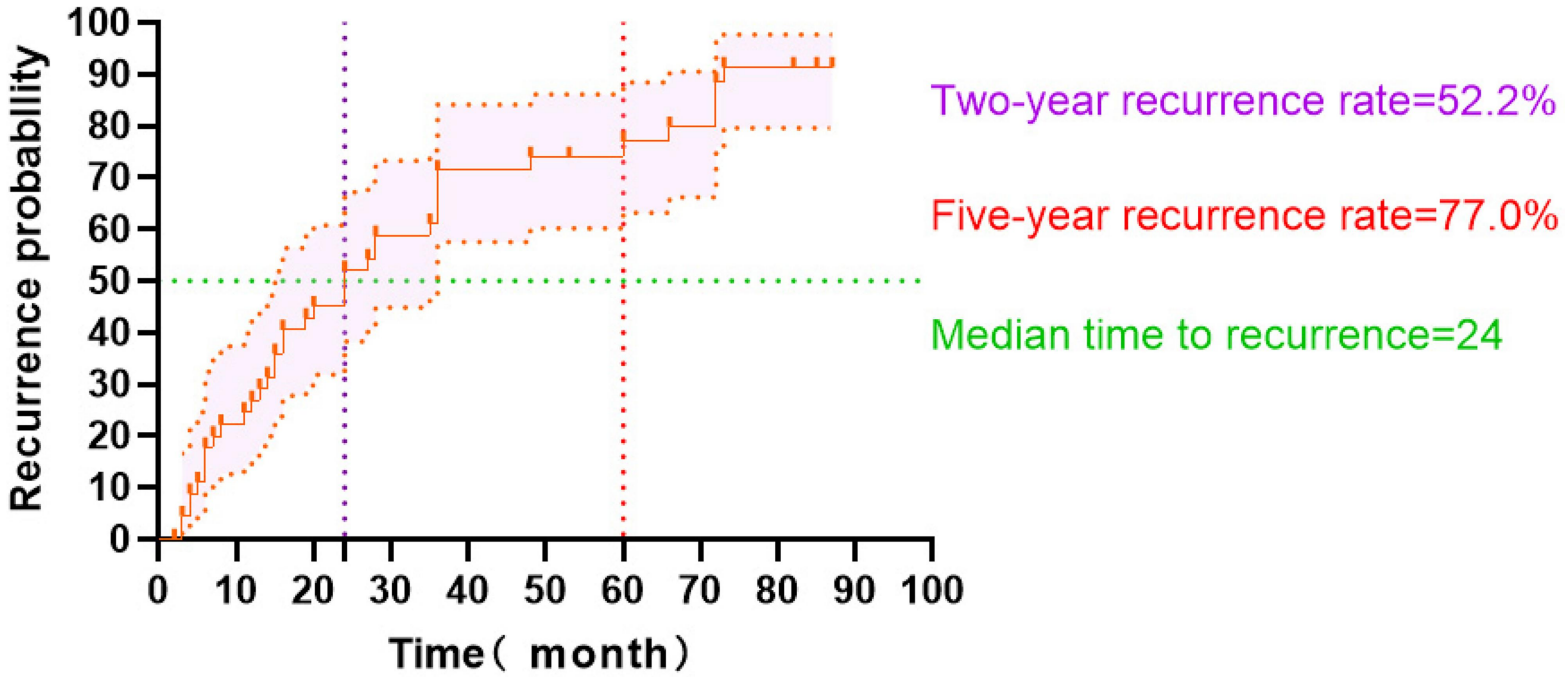
Kaplan–Meier curve showing progression-free survival (PFS) in 46 patients with anaplastic meningioma. The median PFS was 24 months. The 2- and 5-year cumulative recurrence rates were 52.0% and 77.0%, respectively.

Among patients who underwent Simpson Grade I/II resection, postoperative radiotherapy was associated with a numerically prolonged median PFS compared with surgery alone (36 vs. 19 months), although the difference did not reach statistical significance (Mantel–Cox *p* = 0.3155) (Figure 3).

**Figure 3:**
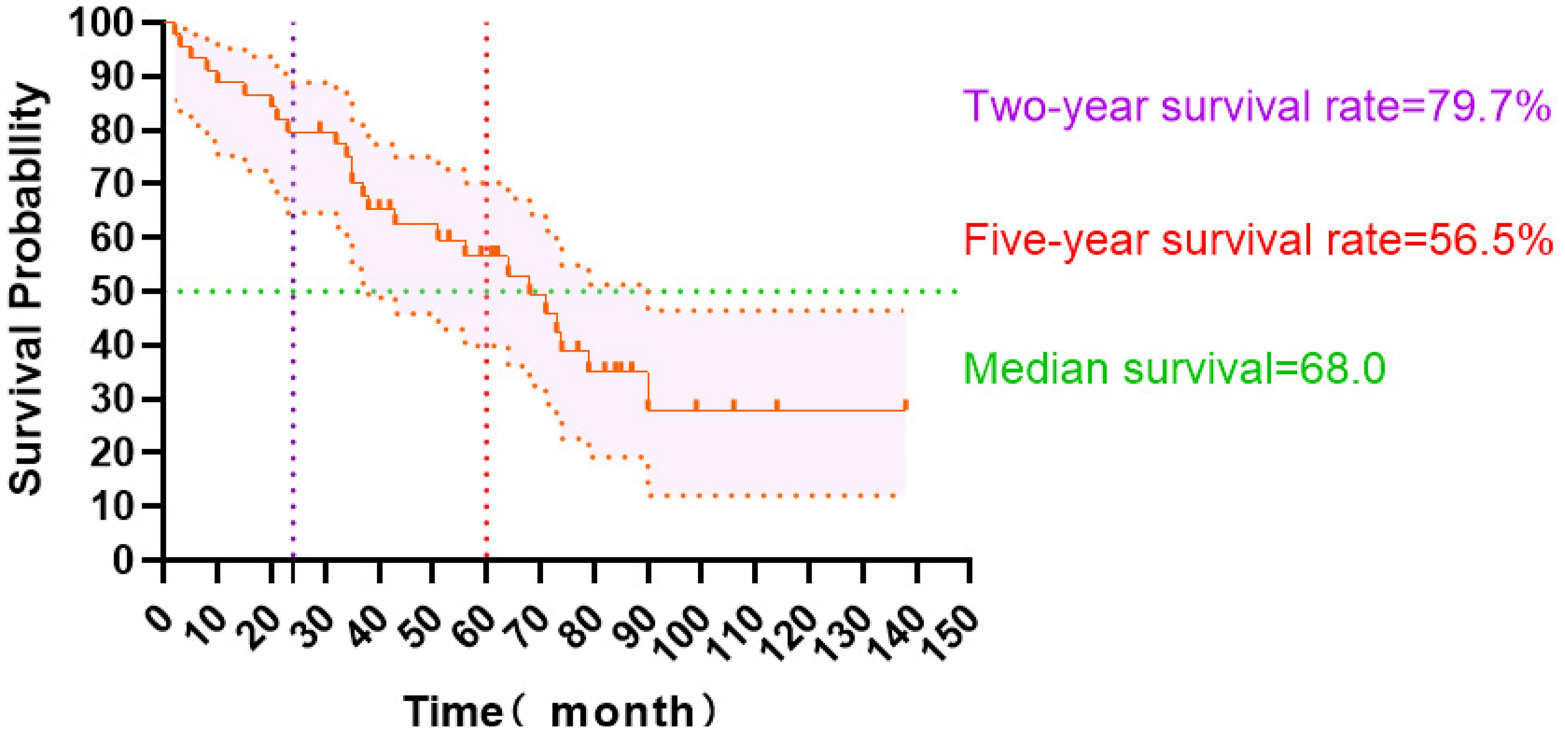
PFS among patients with Simpson Grade I/II resection, comparing those who received RT and those who did not. Median PFS was 36 months in the RT group and 19 months in the non-RT group. Mantel–Cox *p* = 0.3155.

In the Simpson Grade III/IV subgroup, adjuvant radiotherapy significantly delayed early tumor recurrence. The median recurrence-free interval was 15 months in patients receiving radiotherapy compared with 8 months in those without radiotherapy. The Gehan–Breslow–Wilcoxon test demonstrated a significant difference in early recurrence control *p* = 0.0396, whereas no significant difference was observed in long-term recurrence risk using the log-rank test (Mantel–Cox *p* = 0.9152) (Figure 4 and Table 2).

**Figure 4:**
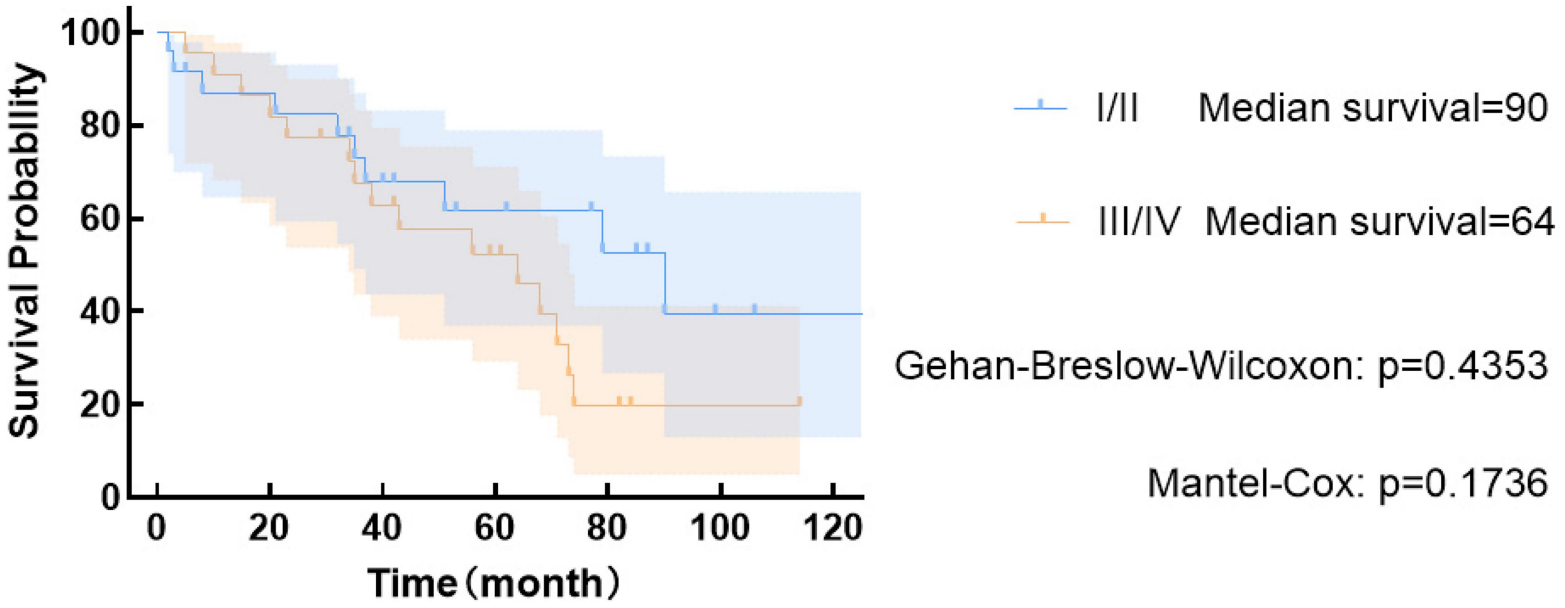
Time to recurrence in patients with Simpson Grade III/IV resection, stratified by radiotherapy. Median recurrence-free interval was 15 months in the RT group vs. 8 months in the non-RT group. Gehan–Breslow–Wilcoxon *p* = 0.0396; Mantel–Cox *p* = 0.9152.

**Table 2:** Progression-free survival according to radiotherapy status.

Subgroup	Median PFS/Recurrence-free interval (months)	*p* value
Simpson I/II + RT	36	0.3155
Simpson I/II without RT	19	
Simpson III/IV + RT	15	0.9152*
Simpson III/IV without RT	8	

* Gehan–Breslow–Wilcoxon test for early recurrence: *p* = 0.0396.

These findings indicate that postoperative radiotherapy may primarily contribute to short-term local disease control rather than durable long-term suppression of recurrence.

### Overall survival

At the time of final follow-up, 25 patients (54.3%) had died. The median overall survival (OS) for the entire cohort was 68 months. The estimated 2- and 5-year OS rates were 79.7% and 56.5%, respectively (Figure 5 and Table 3).

**Figure 5:**
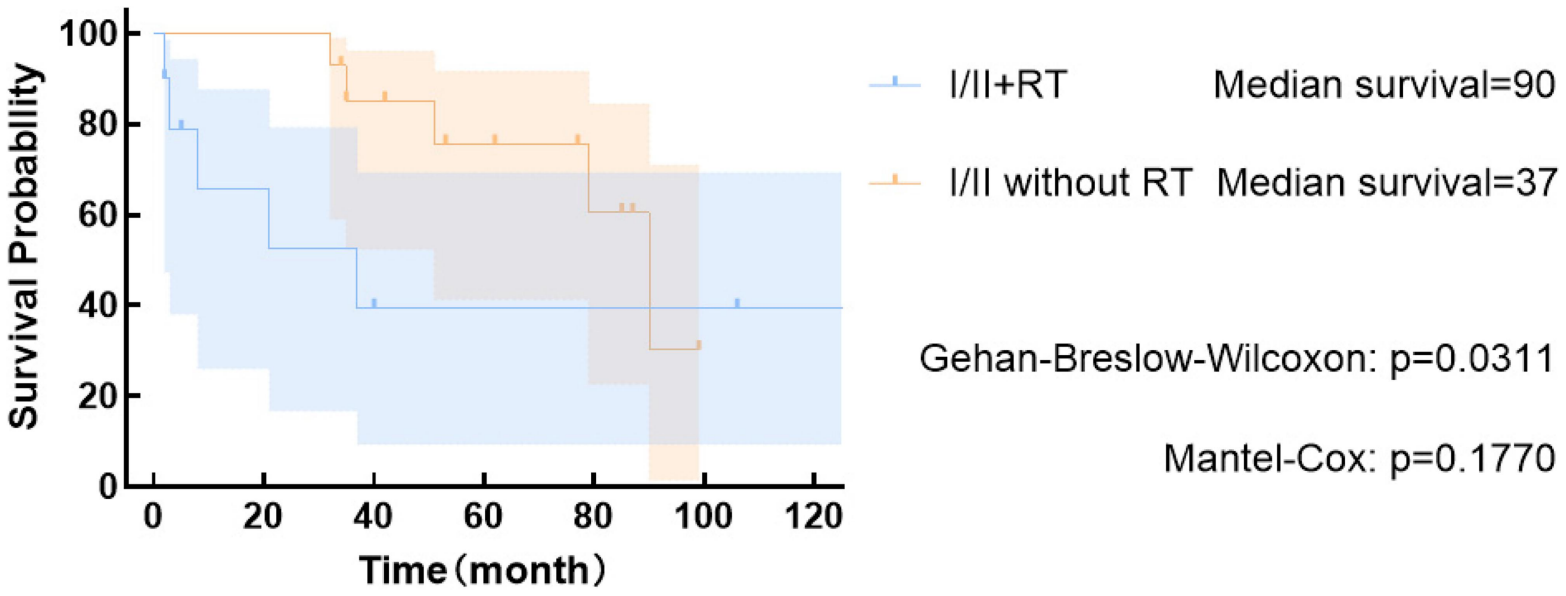
Overall survival (OS) in the entire cohort. The 2-year OS was 79.7%, and the 5-year OS was 56.5%. The median OS was 68 months. The survival curve plateaued after approximately 100 months, but the confidence interval widened, indicating sparse long-term follow-up data.

**Table 3:** Overall survival according to surgical extent and adjuvant radiotherapy.

Variable	Median OS (months)	*p* value
Simpson Grade I/II	90	0.1736
Simpson Grade III/IV	64	
Simpson I/II + RT	90	0.1770*
Simpson I/II without RT	37	
Simpson III/IV + RT	68	0.0596
Simpson III/IV without RT	35	

* Gehan–Breslow–Wilcoxon test: *p* = 0.0311.

Patients who underwent Simpson Grade I/II resection demonstrated longer median OS than those who underwent Simpson Grade III/IV resection (90 vs. 64 months); however, the difference was not statistically significant (Mantel–Cox *p* = 0.1736) (Figure 6).

**Figure 6:**
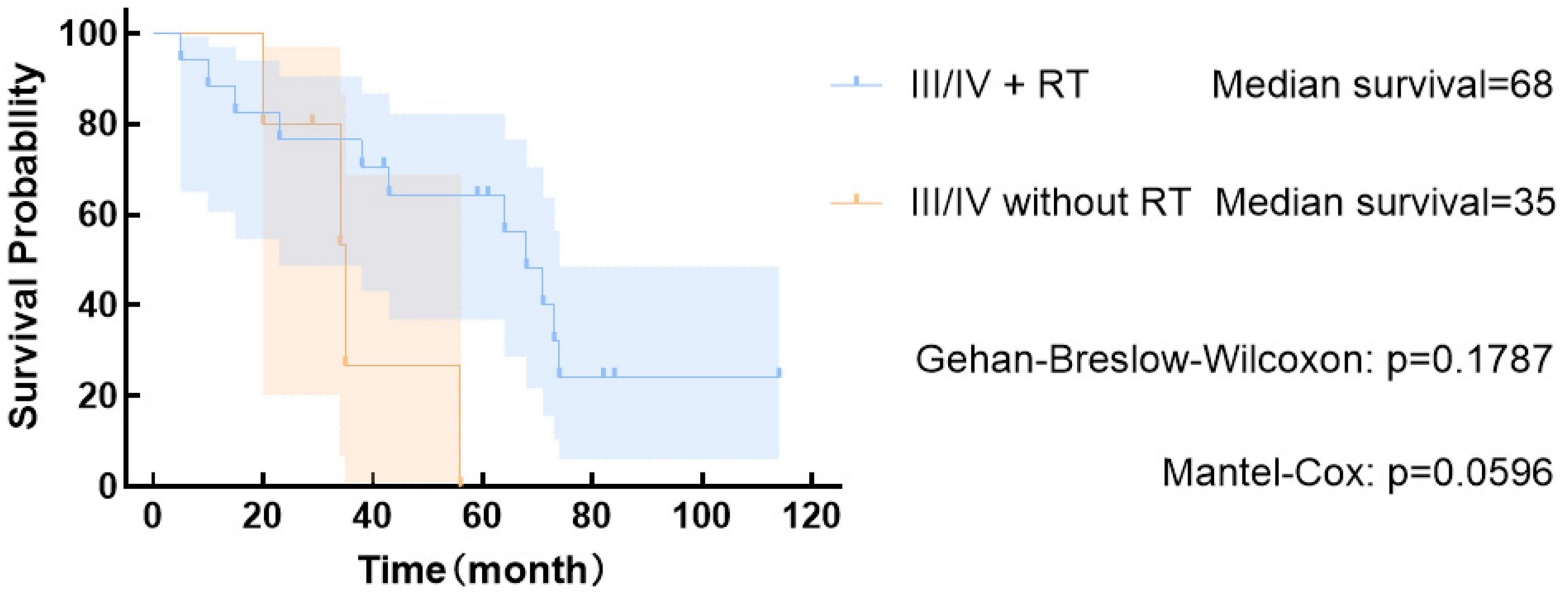
Kaplan–Meier OS curves stratified by extent of resection (Simpson I/II and Simpson III/IV). Patients with Simpson Grade I/II resection had longer OS (median 90 months) compared to those with Simpson Grade III/IV resection (median 64 months) (Mantel–Cox *p* = 0.1736).

Subgroup analysis revealed a trend toward improved survival with postoperative radiotherapy in both surgical groups. In patients with Simpson Grade I/II resection, median OS was 90 months in the radiotherapy group compared with 37 months in the non-radiotherapy group. Although the log-rank test did not demonstrate statistical significance (Mantel–Cox *p* = 0.1770), the Gehan–Breslow–Wilcoxon test suggested a significant early survival benefit associated with radiotherapy *p* = 0.0311 (Figure 7).

**Figure 7:**
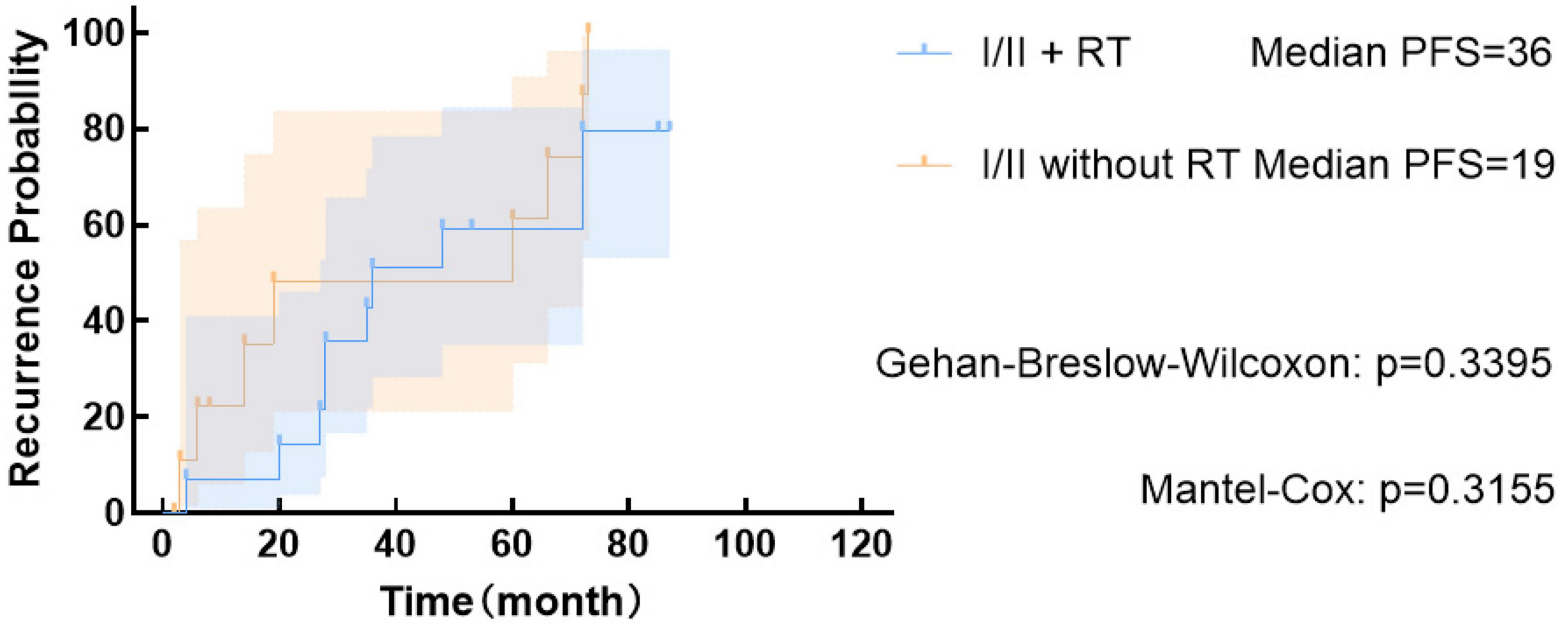
OS in patients with Simpson Grade I/II resection, stratified by receipt of adjuvant radiotherapy (RT). The RT group had significantly longer OS (median 90 months) compared to the non-RT group (median 37 months). Gehan–Breslow–Wilcoxon *p* = 0.0311; Mantel–Cox *p* = 0.1770.

Similarly, among patients with Simpson Grade III/IV resection, median OS was longer in patients receiving radiotherapy than in those without radiotherapy (68 vs. 35 months), with a borderline statistical trend toward significance (Mantel–Cox *p* = 0.0596) (Figure 8).

**Figure 8:**
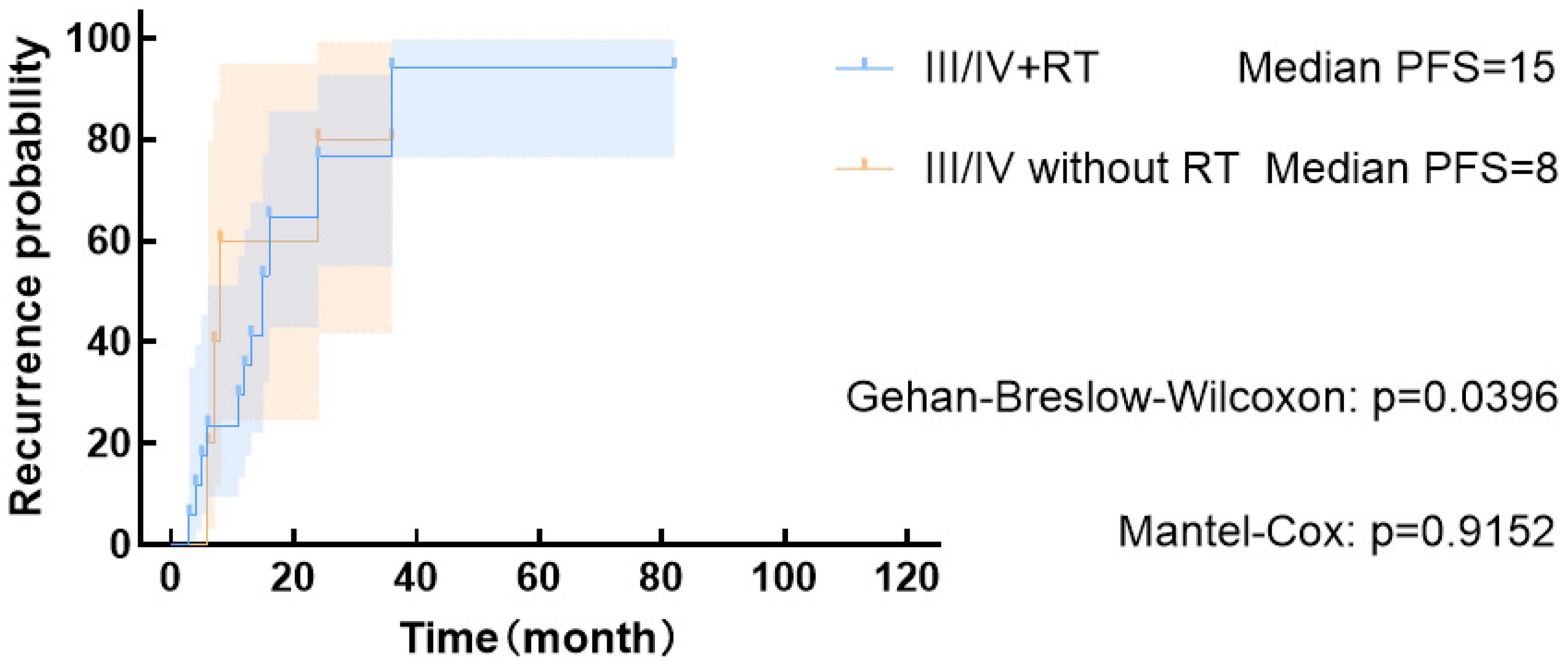
OS in patients with Simpson Grade III/IV resection, with and without adjuvant RT. Median OS was 68 months in the RT group vs. 35 months in the non-RT group. Mantel–Cox *p* = 0.0596.

Overall, these results indicate that aggressive surgical resection combined with adjuvant radiotherapy improves short-term disease control rates and early postoperative survival rates, including PFS; however, this effect appears to be statistically insufficient to significantly alter the long-term overall survival rate in patients with intracranial anaplastic meningiomas.

## Discussion

Anaplastic meningioma is among the most aggressive and therapeutically challenging subtypes of meningioma (11). Although it accounts for only a small percentage of all intracranial meningiomas, its clinical behavior differs notably from that of benign and atypical meningiomas due to its high proliferative activity, infiltrative growth pattern, strong recurrence tendency, and poor long-term survival outcomes (12–14).

In this study, a relatively large cohort of patients with intracranial anaplastic meningioma was retrospectively analyzed, and the relative effects of resection extent and adjuvant radiotherapy on recurrence control and overall survival were evaluated.

Our findings suggest that despite aggressive multimodal treatment, the long-term prognosis remains unfavorable. Tumor recurrence occurred in more than 80% of patients, and the median progression-free survival was only 24 months. Although maximal safe resection and postoperative radiotherapy were associated with improved short-term disease control and delayed recurrence, neither strategy significantly altered the long-term overall survival. These findings further emphasize the intrinsic biological aggressiveness of WHO Grade III meningioma and highlight the limitations of current treatment paradigms.

### Prognostic characteristics and recurrence pattern

Previous research has consistently demonstrated that anaplastic meningiomas are associated with significantly worse outcomes compared to lower-grade meningiomas (15). Reported 5-year survival rates generally range from 30% to 60%, and recurrence rates often exceed 70%, even after gross total resection combined with postoperative radiotherapy (16). Our findings are consistent with these observations, with a 5-year overall survival rate of 56.5% and a recurrence rate of 82.6%.

Notably, in our cohort, recurrence tended to occur early during the follow-up period, particularly within the first two postoperative years. This finding suggests that the early postoperative phase represents a biologically active stage characterized by rapid proliferation of residual tumors and microscopic invasive progression. The tendency towards repeated recurrence also explains why numerous patients required multiple surgical procedures during the course of the disease.

In contrast to benign meningiomas, which often display indolent growth patterns, anaplastic meningiomas appear capable of rapid cellular repopulation even after aggressive cytoreduction (1). This may, in part, account for why improvements in local disease control do not necessarily lead to long-lasting survival benefits.

### Impact of extent of surgical resection

Maximal safe surgical resection remains the fundamental treatment approach for high-grade meningiomas (17). In the present study, patients who underwent Simpson Grade I/II resection had a longer median overall survival than those who underwent Simpson Grade III/IV resection. Although the difference did not reach statistical significance, the observed survival trend validates the importance of achieving maximal cytoreduction whenever anatomically feasible (17).

Nevertheless, the prognostic influence of Simpson grading in anaplastic meningioma appears less significant than in benign meningioma (18). Several factors can explain this phenomenon. Firstly, high-grade meningiomas frequently exhibit microscopic brain invasion, dural infiltration, and irregular growth beyond the radiologically detectable tumor margins. Consequently, even gross total resection may not eliminate the biologically active microscopic disease. Secondly, aggressive attempts at radical resection may be limited by the tumor's involvement of the eloquent cortex, venous sinuses, skull base neurovascular structures, or deep ventricular regions.

The clinical significance of the dural tail sign may also be worthy of consideration in this context. Although traditionally regarded as a characteristic imaging feature of meningioma, dural tail enhancement in anaplastic meningioma may indicate microscopic dural invasion rather than just reactive change. Residual infiltrative disease along the dura may thus result in local recurrence despite an apparently complete resection.

Collectively, these findings suggest that while maximal safe resection remains essential, surgical treatment alone is insufficient to achieve long-term disease control in anaplastic meningioma.

### Role of adjuvant radiotherapy: disease control versus survival benefit

Current clinical guidelines recommend postoperative radiotherapy for World Health Organization (WHO) Grade III meningiomas, irrespective of the extent of surgical resection (9). Nevertheless, the degree of survival benefit conferred by this intervention remains subject to (17, 19) ongoing debate. In the present cohort, adjuvant radiotherapy was correlated with delayed tumor recurrence and prolonged progression-free survival, especially during the early postoperative period (20–22). This effect was most pronounced among patients who underwent subtotal resection, for whom radiotherapy significantly extended the early recurrence-free interval.

Consistent results have been documented in multiple prior retrospective studies and meta-analyses, which have confirmed improved local tumor control following postoperative radiotherapy for high-grade meningiomas (20–23). Notably, however, radiotherapy did not yield a statistically significant improvement in long-term overall survival in this study. This discrepancy between recurrence control and survival benefit can be potentially explained by several biological mechanisms. While radiotherapy may effectively eliminate residual microscopic tumor deposits in the initial phase, treatment-resistant tumor cell clones may eventually emerge as a result of genetic instability, intratumoral heterogeneity, and treatment-induced selection pressure. Furthermore, repeated tumor recurrence progressively restricts the availability of subsequent surgical and radiotherapeutic interventions over time.

A further notable observation is the discrepancy between results obtained from the Gehan–Breslow–Wilcoxon test and the Mantel–Cox log-rank test in certain subgroup analyses. Given that the Gehan–Breslow–Wilcoxon test assigns greater statistical weight to early events, these results indicate that radiotherapy primarily confers early postoperative protection against rapid tumor progression, rather than exerting sustained long-term suppression of tumor recurrence.

These findings further support the hypothesis that existing treatment strategies can delay disease progression without fundamentally modifying the natural biological trajectory of anaplastic meningioma.

The findings of this research indicate that postoperative radiotherapy effectively delays tumor progression and recurrence, especially among patients who have undergone subtotal resection. While the median overall survival was longer in the radiotherapy-receiving cohort, no statistically significant difference was identified in the overall survival profiles between the radiotherapy group and the non-radiotherapy group. Therefore, although postoperative radiotherapy seems to enhance local disease control and may be associated with extended overall survival, this investigation did not detect a statistically significant long-term survival advantage.

These observational results imply that the core clinical benefit of postoperative radiotherapy is the improvement of local tumor control, rather than the definite prolongation of long-term survival. Given the constraints of the limited sample size and the retrospective nature of the study design, the lack of statistical significance cannot be regarded as definitive proof that postoperative radiotherapy provides no therapeutic effect. Larger-scale prospective multicenter studies are needed to determine whether the observed trend of prolonged overall survival represents an actual clinical benefit, and to pinpoint patient subgroups that are most likely to gain advantages from postoperative radiotherapy.

### Biological aggressiveness and molecular characteristics

The highly aggressive clinical behavior observed within our cohort is presumably driven by the distinctive molecular and proliferative characteristics of anaplastic meningioma. A heightened Ki-67 labeling index has persistently been linked to increased mitotic activity, early recurrence, and diminished survival in high-grade meningiomas (24–26). In our research, the mean Ki-67 labeling index reached 47%, corroborating the existence of a highly proliferative tumor phenotype.

In addition to Ki-67, p53 overexpression was detected in a substantial proportion of tumors. Abnormal p53 signaling has been associated with genomic instability and malignant progression in several central nervous system tumors, including anaplastic meningioma. Analogously, elevated expression of transforming growth factor-alpha (TGF-α) may contribute to tumor proliferation, angiogenesis, and invasive behavior.

Recent progress in molecular neuropathology has further deepened the understanding of high-grade meningioma biology. Alterations involving TERT promoter mutation, CDKN2A/B homozygous deletion, BAP1 loss, and DNA methylation subclasses have all been associated with aggressive clinical behavior and poor prognosis. These molecular findings imply that conventional histopathological grading alone may not comprehensively capture the biological heterogeneity of anaplastic meningioma.

Consequently, future prognostic stratification systems will probably necessitate the integration of molecular biomarkers with traditional clinicopathological variables.

### Clinical implications and future directions

The findings of the present study have several notable clinical implications. Firstly, considering that recurrence remains highly prevalent even after intensive multimodal treatment, long-term surveillance through serial contrast—enhanced MRI is essential. Close follow-up appears to be especially critical during the first two postoperative years, when the risk of recurrence is at its peak.

Secondly, repeated surgical intervention may still be requisite for selected patients with recurrent disease. However, the cumulative morbidity associated with repeated craniotomies and repeated irradiation must be meticulously weighed against the potential survival benefit.

Thirdly, our findings underscore the urgent need for more effective systemic therapies. Emerging treatment strategies, such as molecular targeted therapy, anti-angiogenic agents, immune checkpoint inhibitors, peptide receptor radionuclide therapy, and epigenetic modulation, are currently under investigation in recurrent and high-grade meningiomas (27–29). Although clinical evidence is still scarce, these approaches may ultimately provide additional therapeutic options for patients with treatment—resistant disease.

Advanced molecular classification systems may also facilitate individualized therapeutic strategies in the future by identifying biologically distinct subgroups with differential treatment responsiveness (15).

### Limitations

It is necessary to recognize several limitations of this study. Firstly, this was a retrospective study, which inherently carried the risks of selection bias and incomplete data collection. Secondly, the relatively small sample size restricted the statistical power of subgroup analyses and multivariable prognostic modeling. Thirdly, standardized neurological functional outcome measures, such as Karnofsky Performance Status scores, were not consistently accessible throughout the study period. Additionally, molecular profiling data were limited due to the long study interval and the evolving pathological standards.

Despite these limitations, our study represents a relatively large single—study cohort of intracranial anaplastic meningioma with long-term follow-up and offers additional insights into the complex relationship among surgical treatment, radiotherapy, recurrence control, and survival outcome.

## Conclusion

Intracranial anaplastic meningioma is an inherently highly aggressive neoplasm marked by high recurrence rates and unfavorable long-term prognosis (12, 13). Maximal safe resection in combination with postoperative radiotherapy can effectively delay tumor progression and improve local disease control. While the cohort of patients receiving postoperative radiotherapy exhibited longer median overall survival, no statistically significant improvement in overall survival was verified in this study population. These results indicate that the primary clinical benefit of postoperative radiotherapy is the delay of disease progression, rather than providing conclusive evidence of prolonged survival. Larger-scale prospective multicenter studies are required to further elucidate the long-term survival benefit of this treatment modality, and to optimize individualized treatment strategies via molecular risk stratification and newly developed targeted therapies (12, 15).

## Data Availability

The raw data supporting the conclusions of this article will be made available by the authors, without undue reservation.
